# Uncertainty communication, trust and health promotion

**DOI:** 10.24095/hpcdp.45.10.04

**Published:** 2025-10

**Authors:** Jeremy D. Gretton, Angela Mastroianni

**Affiliations:** 1 Behavioural Science Office, Centre for Surveillance, Integrated Insights and Risk Assessment, Data, Surveillance and Foresight Branch, Public Health Agency of Canada, Ottawa, Ontario, Canada; 2 Impact Canada, Impact & Innovation Unit, Privy Council Office, Ottawa, Ontario, Canada

**Keywords:** health communication, public health, uncertainty, risk, trust

## Abstract

Health promotion is more effective when health communicators are considered trustworthy. However, health communicators must often deal with uncertainties in the knowledge base on which they rely. In this commentary, we discuss the benefits of acknowledging uncertainty, with caveats and best practices to cultivate trust. We recommend determining the type of uncertainty involved and selecting appropriate communication approaches. We also advise that communicators emphasize the positive elements of the uncertainty, whenever possible, such as when it reflects a growing evidence base. Health promoters should consider the long-term outcomes of communicating uncertainty, as these may differ from the short-term outcomes. We identify knowledge gaps and areas ripe for future research.

We also show that uncertainty can often be communicated without harming trust in the communicator, and that communicators should rely on evidence-based best practices. We aim to provoke further discussion on how uncertainty should be understood and framed in health promotion efforts, guiding communicators on how to maintain public trust amid unknowns.

**Highlights**

- By leveraging research on uncertainty communication, health promoters can communicate in a manner that helps to foster trust.
- Communicators are advised to keep in mind the specific type of uncertainty they are dealing with.
- Uncertainty may have positive elements, which can be emphasized.
- Whenever possible, consider and assess long-term outcomes of communicating uncertainty.

## Introduction

Health promotion guidelines are more compelling if they come from a trusted messenger.^1^ Communicators can earn trust by conveying information mindfully and transparently, based on the best evidence. Yet health communicators must often address topics that inherently involve uncertainties, such as knowledge gaps or conflicting evidence. Effectively framing uncertainty without eroding trust is a significant challenge. While uncertainty was ubiquitous during the COVID-19 pandemic, it continues to affect health promotion research and guidelines in areas such as exercise, nutrition and vaccination.

The aim of this commentary is to provide insight into when uncertainty leads to trust or mis/distrust, and to provide health communicators with evidence-based approaches for conveying uncertainties in ways that cultivate trust. We emphasize that the effects of uncertainty on trust depend on various factors, including how it is communicated, messenger credibility and the type of uncertainty involved. Drawing upon previous reviews,^2^ recent and relevant academic literature^3^,^4^ and our academic and public health experiences, we offer recommendations acknowledging nuances and complexities. We highlight the limitations of previous research and suggest areas for further work.

We recommend that health communicators (1) determine the type of uncertainty involved and select appropriate communication tactics; (2) normalize uncertainty while maintaining accuracy; and (3) consider long-term outcomes of communicating uncertainty.

## Best practices when communicating uncertainty

***1. Determine the type of uncertainty involved and select appropriate communication tactics***

Uncertainty is inherent to science and comes in many forms,^2^ with each having implications for the audience’s response. We discuss three types of specific categories or forms of uncertainty, and highlight links with mis/distrust and suggest areas for further research:

- Deficient uncertainty: A known knowledge gap.
- Consensus uncertainty: Disagreement among people or data sources.
- Technical uncertainty: Numeric uncertainty such as margins of error.

**Deficient uncertainty**

Communicating unknowns can foster or hinder trust.^2^ In this subsection, we focus on one common way of communicating knowledge gaps: hedging. In keeping with previous research, we distinguish between discourse-based hedging and lexical hedging.

Discourse-based hedging refers to acknowledging limitations or caveats, such as noting that a study result might not be reliable because of its small sample size.

In one study, college students read one of five news articles about cancer, for example, whether lycopene consumption can prevent prostate cancer.^5^ The articles included high or minimal levels of discourse-based hedging, attributed to the primary or an unaffiliated researcher. When the primary scientist hedged, they—and the journalists who wrote the article—were rated as more trustworthy. Ratings of expertise were not affected. When the study was replicated with participants recruited in shopping malls reading four news articles,^6^ hedging by the primary scientist was associated with higher journalist—but not scientist—credibility ratings compared with a low-uncertainty condition. Thus, when presenting research findings, hedging may enhance, or at least not reduce, trustworthiness.

Given the inconsistent effects of discourse-based hedging on audiences’ perceptions of scientists, future research should explore moderating conditions, such as whether discourse-based hedging is more accepted by audiences with more formal education.^7^,^8^

Discourse-based hedging has also been examined in the context of COVID-19. Hedging by a scientist—including expressions of uncertainty due to limited data or other reasons affecting their estimate of the prevalence of post COVID-19 condition (long COVID)—was associated with less trust in that scientist compared with when the scientist did not hedge.^3^ However, the scientist’s degree of uncertainty might have been so strong that it elicited distrust; they stated that “the study result of 13% is of limited significance” [translated from German]. In addition, measures of scientist integrity, benevolence and competence were unaffected. Hedging regarding hypothetical COVID-19 vaccine side effects or efficacy has been shown to not influence trust initially, and to even buffer trust to an extent, in the face of changing evidence (see best practices recommendation 3, “Consider long-term outcomes of communicating uncertainty”).^9^,^10^ With some exceptions,^11^,^12^ health-related discourse-based hedging does not appear to diminish trust,^13^ may increase trust^14^ and may be beneficial for transparency. Since research on hedging sometimes incorporates additional types of uncertainty, such as technical uncertainty,^9^ future work should aim to further clarify unique effects of deficient uncertainty.

Lexical hedges include words or phrases such as “might” and “could.”^5^ Some studies found that lexical hedges did not affect trust in the sources that made claims about cancer, vaccines, mask-wearing (preventing coronavirus transmission) or other topics.^14^-^16^ In another study, Durik et al. reported that colloquial lexical hedges (e.g. “sort of”), but not professional ones (e.g. “may”), were associated with more negative impressions of a communicator compared with the absence of hedges.^17^ However, this was only the case among participants with lower scientific reasoning scores. Thus, for lexical hedging, words may matter—and formality in health promotion messaging might be beneficial.

Promising future research directions include clarifying the impacts of other qualities of hedges, including extremity, that is, whether hedges temper a claim or negate it altogether.

**Consensus uncertainty**

Consensus uncertainty is often received negatively.^2^ Reading conflicting research findings about jogging or milk consumption can foster more negative attitudes toward health research.^18^ Likewise, conflicting messages about whether red meat consumption causes cancer, involving disagreement among researchers or differences between findings, reduced trust in scientists compared to a consistent-findings control condition. This was pronounced when the scenario involved researcher or evidence disagreement rather than another scenario involving changing guidance from the same source.^19^

These findings suggest that health communicators might benefit from presenting a united front when there is genuine agreement. In these situations, communicators may also maintain trust by avoiding perceptions of collusion, particularly for skeptical audiences. Aklin and Urpelainen found that greater expert consensus enhanced policy support among people who trusted scientists, but reduced it among those who distrusted scientists—potentially because it implied collusion.^20^ Future research may help elucidate how to emphasize consensus without the appearance of collusion. 

Amid consensus uncertainty, precautionary language may sometimes enhance trust. After reading about consensus uncertainty concerning a fictitious health risk (a micro-organism in tap water), participants in a Canadian study reported marginally higher trust in the government when that government presented the situation as a potential risk and recommended precautions.^21^ Any discussion of precautionary approaches can be informed by an understanding of audiences’ values and costs and benefits of precautions.^22^

**Technical uncertainty**

Technical uncertainty is associated with positive or neutral effects on credibility and other outcomes,^2^ though some negative effects have been reported.^23^,^24^

When describing numbers, expressing technical uncertainty in words (e.g. by saying, “There is some uncertainty around this estimate”) may lead to greater distrust of the numbers and the source compared to numeric uncertainty (e.g. by providing a range) or not acknowledging uncertainty.^25^,^26^ As with hedging, the specific wording might matter: advisers are at times perceived more negatively when they use the word “probably.”^27^ The effects on trust of verbalizing technical uncertainty appear to be relatively small.^25^,^27^

When expressing technical uncertainty using numeric ranges, providing guidance as a narrow range may be better received than if this is a wider range.^27^ For example, people were more likely to rely on others’ estimates (e.g. regarding calories in food items) when these were provided as low-uncertainty ranges, rather than wider ranges or point estimates.^28^

Risk presentation also affects the messenger’s credibility. When presenting risks of an acne medication’s side effects, the messenger was seen as less credible when presenting a range rather than a point estimate.^24^ Of note, if the range was relatively narrow, credibility was spared when the messenger was a hypothetical local primary care clinician (versus a hypothetical pharmaceutical company).^24^ Exploring synergies between messenger credibility and uncertainty acknowledgement with regards to trust is another promising avenue for future research.

***2. Normalize uncertainty while maintaining accuracy***

Researchers have examined how to “normalize” uncertainty,^29^ emphasizing that uncertainty is expected or desirable as a part of the scientific process in order to make it more acceptable. In the following subsections, we group findings based on whether this framing occurs before, during or after the communication of uncertainty.

**Normalizing uncertainty before communicating uncertainty**

Pre-emptively framing uncertainty positively can protect credibility. Although reminders of changes or inconsistency in COVID-19 data and guidance (e.g. about wearing masks) can diminish experts’ credibility, Gretton et al. found that this may be mitigated by pre-emptively emphasizing that change is expected and indicates scientific progress.^4^ Likewise, reading about the evolving nature of science resulted in people having more positive attitudes toward science when receiving conflicting messages about carbohydrate or alcohol consumption, mammography or prostate-specific antigen testing.^30^ It is unclear, however, if the framing helped improve receptiveness to uncertainty or to science in general, because the study lacked a “no-uncertainty” control condition.^30^ Both studies presented consensus uncertainty indirectly (e.g. via hypothetical people on social media), meaning the direct application for health communicators is unclear.

Similarly, if people were shown climate change projections as ranges after reading that science should be characterized by debate and uncertainty, they were more likely to express pro-environmental behavioural intentions than if they were first told that science seeks absolute truth.^31^ This suggests that framing uncertainty as fundamental to science can make uncertainty more acceptable, though further research in health contexts is needed.

**Normalizing uncertainty while communicating uncertainty**

In one study, information about a hypothetical H7N3 flu outbreak and vaccine was presented to participants in Spain using certain language, uncertain language only or uncertain language paired with normalizing language (e.g. “In life, we never have perfect knowledge of any health risks…”).^29^ The messenger was ostensibly the director of the Ministry of Health. Trust ratings for this ministry were lower amid uncertainty, even if normalized. Although similar studies have been conducted,^11^ to our knowledge they examined the existence of uncertainty rather than the communication of uncertainty by a messenger.

Research on simultaneous uncertainty-normalization is quite limited. Furthermore, pre-emptive normalization is not always possible. Additional research into the normalization of uncertainty while (or after) communicating uncertainty could be beneficial.

***Normalizing uncertainty after communicating uncertainty***

Lyons et al. found that providing an uncertainty-normalizing message after a change in recommended antibiotic regimens did not affect the rated credibility of medical experts or doctors.^32^ However, the changing (versus consistent) guidance did not affect credibility in the first place, and the manipulation check was not significant for the brief uncertainty-normalizing intervention. As a result, we are hesitant to generalize beyond this study.

Other studies provide evidence in favour of uncertainty-normalizing messages following uncertainty communication. Flemming et al. noted that although there is often a negative association between the perceived tentativeness of findings reported in an article and that article’s rated credibility, this relationship can be neutralized by subsequently sharing a message arguing for the acceptability of research results being tentative.^33^ However, tentativeness would need to be experimentally manipulated to determine if it causes these effects on credibility.

In addition to examining the effects of timing of normalization, future research could clarify the roles of messengers. Normalization might be more effective if provided by a distinct source rather than by the messenger who acknowledges the specific uncertainty.

***3. Consider long-term outcomes of communicating uncertainty***

It is important to assess short- and long-term responses to communicating uncertainty. For example, Batteux et al. reported that communicating uncertainty about the efficacy of COVID-19 vaccines does not necessarily reduce trust initially.^10^ Following evidence of lower vaccine efficacy than previously stated, trust in a government representative generally diminished—but less so if people had initially received a message that conveyed uncertainty versus one that expressed greater certainty.^10^ Other research also suggests that initial uncertainty can make negative news more acceptable.^9^,^34^ However, yet other studies indicate that a numeric estimate—uncertain or otherwise—might make bad news more palatable than an initial verbal statement (e.g. “unlikely”).^35^

We are not aware of research exploring whether repeated communication of uncertainty over time affects trust, though some have proposed examining cumulative effects.^36^ Such research could be valuable given that uncertainty often takes time to resolve.

## Conclusion

For health communication to be transparent, mentioning uncertainty is necessary—but distrust is not. In this commentary we offer actionable strategies—categorizing uncertainty, normalizing it and considering long-term outcomes of communicating it—that health promoters can leverage to improve uncertainty communication. Applying evidence-based messaging strategies can promote trust and encourage the uptake of health promotion guidelines. Amid many unknowns, that much is certain.

## Acknowledgements

We would like to thank Gabriela Capurro, Catherine Guo, Mark Morrissey, Rhiannon Mosher, Lucie Plja, Gabrielle Plamondon and Bethany Simison, of the Public Health Agency of Canada, and Kieran Findlater and Klajdi Puka, of the Privy Council Office of the Government of Canada, for their contributions to the initial conceptualization and/or review of previous versions of this commentary. This work was supported by and has benefited from the partnership between the Privy Council Office’s Impact Canada and the Public Health Agency of Canada’s Behavioural Science Office.

## Funding

None.

## Conflicts of interest

The authors declare no competing interests.

## Authors’ contributions and statement

JG, AM: Conceptualization, investigation, writing—original draft, writing—review and editing.

The content and views expressed in this article are those of the authors and do not necessarily reflect those of the Government of Canada.
